# Impact of the COVID-19 Pandemic on Presentation, Microbiology, and Outcomes of Infective Endocarditis: A Romanian Retrospective Cohort Study

**DOI:** 10.3390/antibiotics15080786

**Published:** 2026-08-14

**Authors:** Adelina Maria Radu, Irina Florentina Talpoși, Raluca Elena Țoțoiu, Roxana Berbecaru, Ana Dobrin, Violeta Melinte, Cristina Maria Văcăroiu, Tiberiu Sebastian Holban, Matei Cherecheanu Popa, Oana Andreea Popa, Mădălina Elena Iancu, Amalia Călinoiu, Luminița Tomescu, Oana Săndulescu, Valeriu Gheorghiță

**Affiliations:** 1 Department of Infectious Diseases, “Carol Davila” University of Medicine and Pharmacy, 050474 Bucharest, Romaniaana.dobrin@drd.umfcd.ro (A.D.); dr.melinte@gmail.com (V.M.); matei.cherecheanu@gmail.com (M.C.P.); oana_noa@yahoo.com (O.A.P.); luminita.tomescu@umfcd.ro (L.T.); valeriu.gheorghita@umfcd.ro (V.G.); 2 Agrippa Ionescu Clinical Emergency Hospital, 011356 Bucharest, Romania; raluca-elena.totoiu@rez.umfcd.ro (R.E.Ț.); roxana-oana.berbecaru0925@rez.umfcd.ro (R.B.); cristinamarv@yahoo.com (C.M.V.); tsh0912@gmail.com (T.S.H.); madalina.iancu@gmail.com (M.E.I.); acalinoiu@gmail.com (A.C.); 3 “Prof. Dr. Matei Balș” National Institute for Infectious Diseases, 021105 Bucharest, Romania

**Keywords:** infective endocarditis, COVID-19, *Enterococcus*, *Staphylococcus*, embolism, mortality

## Abstract

Objectives: This study aimed to assess the impact of the COVID-19 pandemic on the epidemiology, microbiology, clinical presentation, management, and outcomes of infective endocarditis (IE). Methods: A retrospective cohort study was conducted, including 100 adult patients with definite IE diagnosed between January 2020 and December 2025 at a Romanian tertiary care centre. Patients were stratified by year of admission into pandemic (2020–2022) and post-pandemic (2023–2025) groups. Results: Among the 100 patients (47 in the pandemic period and 53 in the post-pandemic period), the time from symptom onset to diagnosis was significantly shorter in the post-pandemic period (median of 24 days versus 40 days; *p* < 0.01). We observed changes in the microbiological profile. The proportion of staphylococcal IE was lower, while the proportions of enterococcal and streptococcal IE were slightly higher in the post-pandemic period. In-hospital mortality decreased significantly from 51.1% to 24.5% (*p* = 0.006). Embolic events were more frequent in the post-pandemic period (62.3% vs. 39.1%; *p* = 0.022). Vegetation size was associated with embolic risk (OR: 1.05 per mm; *p* < 0.01). Pandemic-period admission (aOR 3.11; *p* = 0.020) and sepsis (aOR 11.30; *p* = 0.006) were independently associated with higher in-hospital mortality. Conclusions: The COVID-19 pandemic was associated with delayed presentation and worse outcomes in IE. Post-pandemic improvements likely reflect healthcare system adaptation and earlier diagnosis. Microbiological aetiology remains a key determinant of prognosis.

## 1. Introduction

Infective endocarditis (IE) is a severe disease associated with high morbidity and mortality, with an estimated incidence of 3–10 cases per 100,000 population and mortality rates of 20–30% [1]. The epidemiology of IE has evolved substantially over recent decades due to population ageing, increasing comorbidity burden, and greater exposure to healthcare-associated interventions. These factors have contributed to a rising incidence and a shift toward more complex, high-risk patient profiles [2].

IE is associated with significant short- and long-term mortality and frequent complications. Outcomes are influenced by patient characteristics, access to specialised care, and, importantly, the causative microorganism. Delayed diagnosis and limited access to multidisciplinary endocarditis teams are associated with worse outcomes, including higher complication rates and delayed use or underuse of indicated cardiac surgery [3].

The microbiological profile of IE has also changed. While viridans group streptococci historically predominated, staphylococci—particularly *Staphylococcus aureus*—are now leading pathogens in many contemporary series, reflecting the increasing burden of healthcare-associated infections [2,3,4]. In addition, enterococci, especially *Enterococcus faecalis*, are increasingly recognised, particularly in elderly patients and those with prior healthcare exposure. Microbial aetiology is a major determinant of disease severity and prognosis [2,5].

Advances in diagnostic techniques and treatment strategies, together with multidisciplinary team approaches, have improved outcomes [6,7,8]. However, the SARS-CoV-2 pandemic placed unprecedented strain on healthcare systems, disrupting diagnostic pathways, delaying presentation, and limiting access to specialised care. A previous report has described reductions in IE diagnoses and cardiac surgical procedures during peak pandemic periods, although the long-term consequences remain unclear [9].

Data from Eastern Europe remain limited, particularly regarding the impact of the pandemic on IE. This study aimed to evaluate changes in epidemiology, microbiology, management, and outcomes of IE in a Romanian tertiary care centre during and after the COVID-19 pandemic.

## 2. Results

A total of 100 patients with definite infective endocarditis were included: 47 during the pandemic and 53 afterward. The baseline characteristics of the two groups were comparable (Table 1), including age (median 68 vs. 67 years, *p* = 0.853), sex distribution, and type of valve involvement.

**Figure 1:**
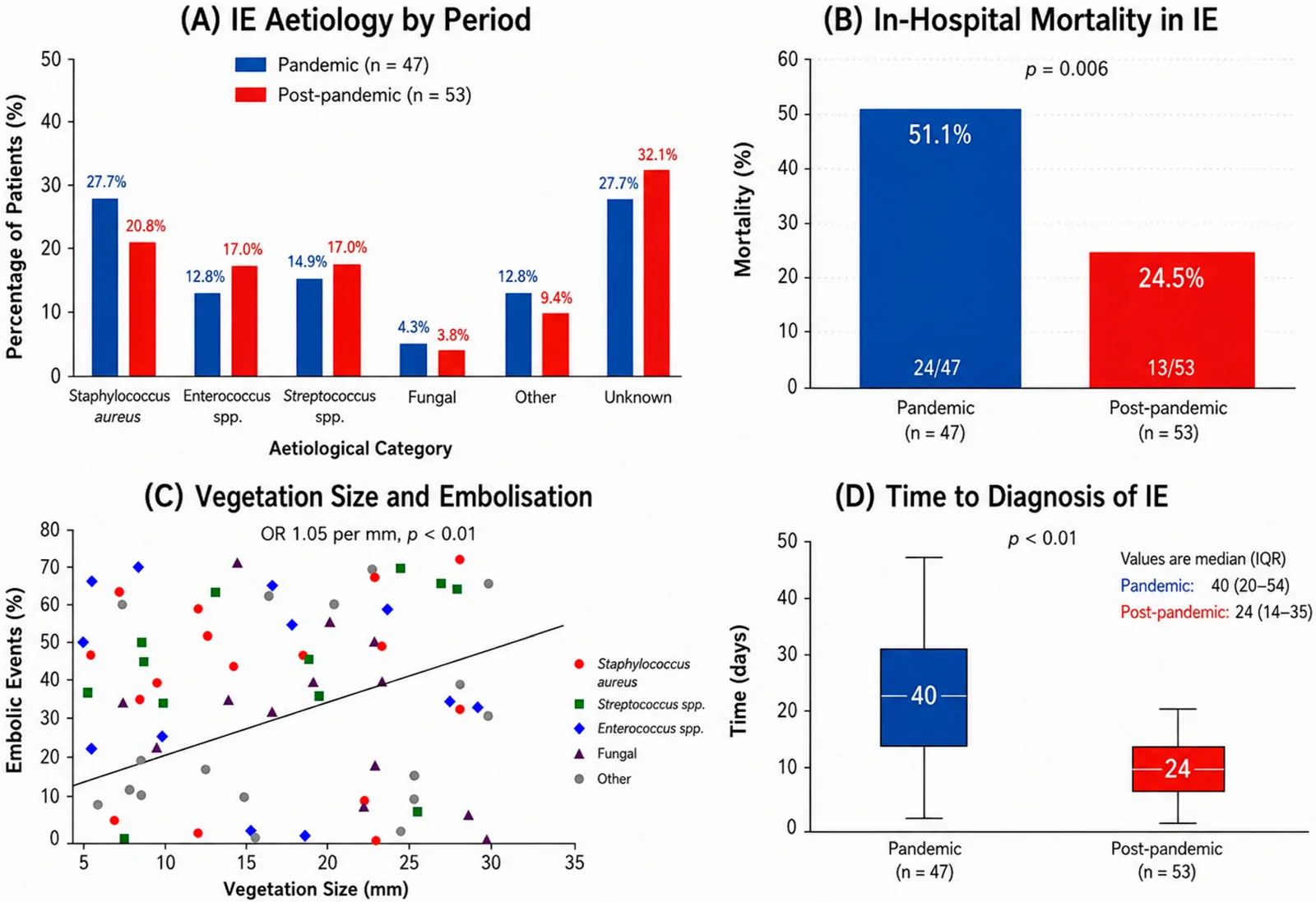
Microbiological distribution, outcomes, and diagnostic parameters of infective endocarditis during the pandemic and post-pandemic periods. (**A**) Aetiological distribution by study period. (**B**) In-hospital mortality (%), higher during the pandemic (51.1%) compared with the post-pandemic period (24.5%) (*p* = 0.006). (**C**) Relationship between vegetation size and embolic events, showing increased embolic risk with larger vegetations (OR 1.05 per mm, *p* < 0.01). (**D**) Time to diagnosis (days), shorter in the post-pandemic period (median 24 vs. 40 days, *p* < 0.01). Data are presented as percentages or medians, as appropriate. Abbreviations: IE, infective endocarditis; OR, odds ratio.

The microbiological profile showed descriptive differences between the two periods (Figure 1A). There was a reduction in staphylococcal IE (27.7% vs. 20.8%) and a relative increase in enterococcal (12.8% vs. 17.0%) and streptococcal (14.9% vs. 17.0%) cases post-pandemic. Culture-negative cases were more frequent post-pandemic (Table 1).

Embolic events were more frequent in the post-pandemic period (39.1% vs. 62.3%, *p* = 0.022), particularly central nervous system embolism (6.4% vs. 30.2%, *p* = 0.002). Perivalvular abscess was observed in 14.9% of pandemic and 22.6% of post-pandemic patients (*p* = 0.324). Vegetation size was significantly associated with embolic complications (Figure 1C). In logistic regression analysis, each 1-mm increase in vegetation size was associated with a 5% increase in the odds of an embolic event (OR 1.05 per mm, *p* < 0.01), supporting vegetation size as an important marker of embolic risk.

The time to diagnosis was significantly shorter post-pandemic (median 40 vs. 24 days, *p* < 0.01; Figure 1D), though the duration of antibiotics and length of hospital stay were similar (Table 1). In univariable logistic regression analysis, admission during the pandemic period was associated with significantly higher odds of in-hospital mortality compared with the post-pandemic period (OR 3.21, 95% CI 1.38–7.49; *p* = 0.007). Longer diagnostic delay was also associated with mortality, with a 15% increase in the odds of in-hospital death for each additional 10 days from symptom onset to hospital admission (OR 1.15, 95% CI 1.02–1.29; *p* = 0.027). The presence of sepsis criteria was associated with substantially increased mortality (OR 8.57, 95% CI 1.71–43.02; *p* = 0.009). In a multivariable model including study period, diagnostic delay, and sepsis, pandemic-period admission remained independently associated with higher in-hospital mortality (adjusted OR 3.11, 95% CI 1.20–8.08; *p* = 0.020), as did the presence of sepsis criteria (adjusted OR 11.30, 95% CI 2.01–63.41; *p* = 0.006). Diagnostic delay was no longer statistically significant after adjustment (adjusted OR 1.11 per 10 days, 95% CI 0.97–1.26; *p* = 0.138).

In-hospital mortality was significantly higher during the pandemic (51.1% vs. 24.5%; *p* = 0.006; Figure 1B), resulting in lower survival rates (48.9% vs. 75.5%; *p* = 0.006; Table 1). Recurrence rates were low and similar between groups. In univariable logistic regression analysis, admission during the pandemic period was associated with approximately three-fold higher odds of in-hospital death compared with admission during the post-pandemic period (OR 3.21, 95% CI 1.38–7.49; *p* = 0.006). These findings indicate a significant association between study period and in-hospital mortality.

All in-hospital deaths in both study periods were directly attributable to infective endocarditis or its immediate complications; no deaths were attributed to COVID-19 infection, unrelated comorbid conditions, or other pandemic-specific causes.

## 3. Discussion

This study identified significant changes in the presentation, microbiology, and outcomes of infective endocarditis (IE) during and after the pandemic. These results underscore the impact of healthcare system disruption during the pandemic and the subsequent recovery of diagnostic and management processes.

One notable finding is the substantial reduction in time to diagnosis during the post-pandemic period. This likely reflects improved access to healthcare, restored referral pathways, and increased clinical awareness following the pandemic. During the pandemic, delayed presentation and restricted access to care likely contributed to prolonged symptom duration prior to diagnosis [9]. Although diagnostic timeliness improved post-pandemic, it did not fully translate into reduced disease severity at presentation.

Despite earlier diagnoses, post-pandemic patients experienced more frequent embolic events, particularly central nervous system embolism, while perivalvular abscesses were numerically more common [10,11]. This may be due to several factors. Differences in pathogen virulence, vegetation characteristics, and host comorbidities may influence the risk of embolization independently of diagnostic timing. Second, more widespread use of advanced cardiac imaging, particularly TEE, after the pandemic may have led to more cases of abscesses being detected. Finally, embolic complications often occur before infective endocarditis is suspected, meaning that earlier diagnosis after hospital presentation may not prevent these events. Therefore, diagnostic delay is just one of several factors determining disease severity.

The significant association between vegetation size and embolic events is clinically relevant. Each 1-mm increase in vegetation size was associated with a 5% increase in the odds of embolism, suggesting a progressive increase in embolic risk with increasing vegetation burden. This finding reinforces the importance of careful echocardiographic assessment of vegetation size when evaluating embolic risk and considering the timing of multidisciplinary and surgical management [11,12,13].

We also observed descriptive changes in microbiological aetiology: a lower proportion of staphylococcal infections and slightly higher proportions of enterococcal and streptococcal IE post-pandemic. This pattern is consistent with evolving epidemiological trends and may reflect changes in healthcare exposure, patient demographics, and the resumption of elective procedures [4,12,14,15].

The persistently high proportion of culture-negative cases underscores the ongoing limitations of microbiological diagnostics and the necessity of improved techniques, including molecular methods [6,16].

In-hospital mortality was significantly higher during the pandemic, which underscores the impact of healthcare disruption. Although the deaths were directly IE-related, pandemic-related healthcare disruption may have indirectly contributed to mortality through delayed presentation, delayed diagnosis, more advanced disease at admission, and possible delays in access to specialised or surgical management. The substantial improvement in survival post-pandemic underscores the importance of timely diagnosis, multidisciplinary management, and access to cardiac surgery [11,17].

These findings suggest that improving diagnostic timeliness alone is insufficient to optimise outcomes. Disease severity at presentation, microbial aetiology, and access to specialised care remain key determinants of prognosis [8,15,18,19].

This study has limitations, including its retrospective, single-centre design and moderate sample size. An additional limitation is the absence of a pre-pandemic comparator cohort from the same center. Potential changes in referral patterns may have introduced selection bias as well. In addition to the limitations inherent in its retrospective, single-centre design, this study did not systematically evaluate changes over time in the use, timing or frequency of transoesophageal echocardiography (TEE). The increased availability of TEE or its earlier implementation in the post-pandemic period may have improved the detection of vegetations and perivalvular complications, thereby influencing abscess formation and other imaging findings. Furthermore, we did not assess the evolution of blood culture-negative infective endocarditis over the study period, nor did we consider the use of molecular techniques, prolonged culture incubation, serological testing for fastidious organisms or 16S rRNA sequencing. These factors may have impacted the microbiological diagnosis and must be considered when interpreting changes in pathogen distribution between the study periods.

Nevertheless, the study provides valuable data from Eastern Europe, a region that is underrepresented in IE research.

## 4. Materials and Methods

### 4.1. Study Design and Setting

This retrospective observational cohort study was conducted at a university hospital providing tertiary care in Bucharest, Romania. The study adhered to the Strengthening the Reporting of Observational Studies in Epidemiology (STROBE) guidelines.

### 4.2. Study Population

**Figure 2:**
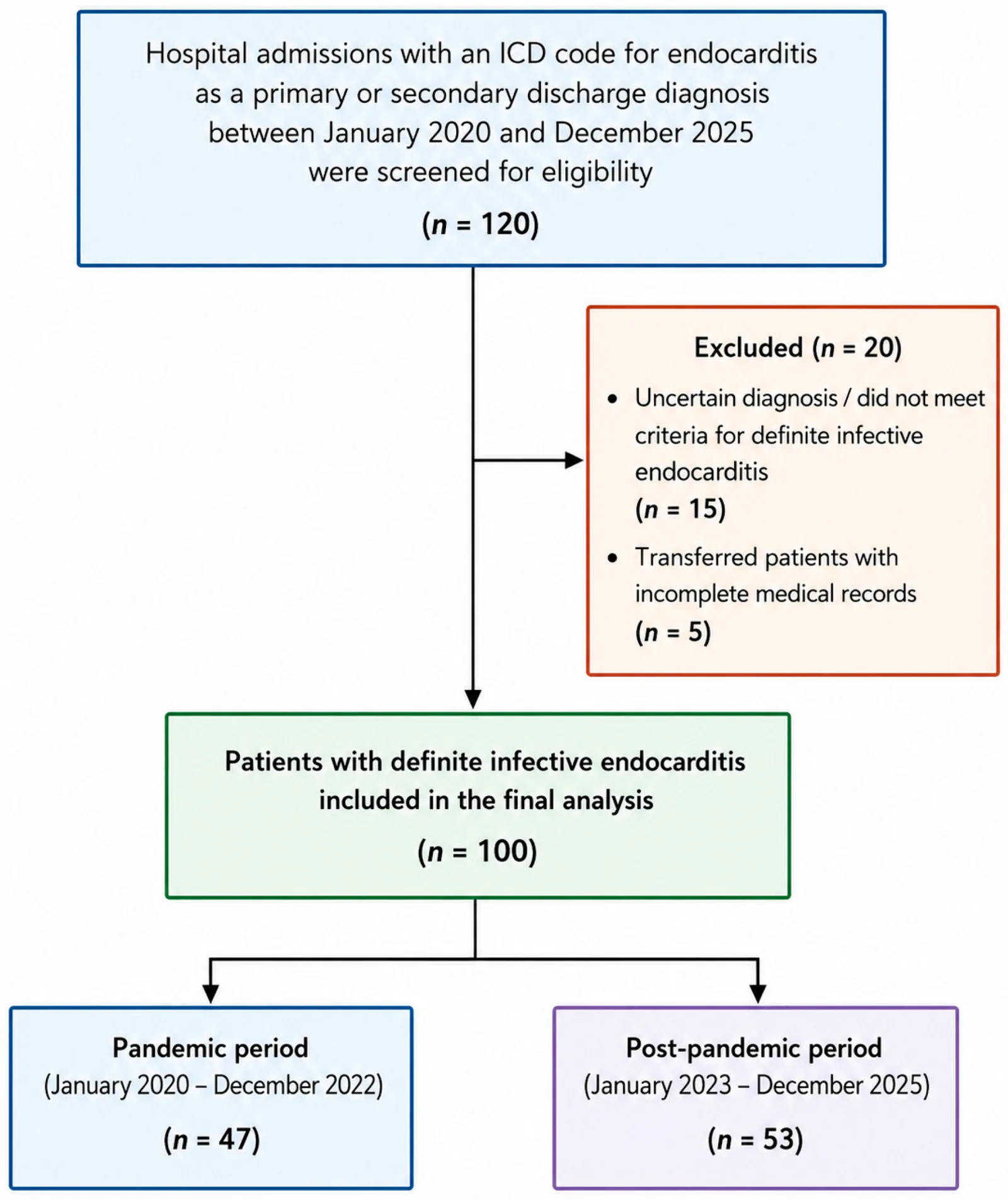
Patient-selection flow diagram. Hospital admissions with an ICD code for endocarditis as a primary or secondary discharge diagnosis between January 2020 and December 2025 were screened for eligibility. Of 120 cases screened, 20 were excluded: 15 because the diagnosis was uncertain or did not fulfil the criteria for definite infective endocarditis and five transferred patients because of incomplete medical records. The final cohort comprised 100 patients with definite infective endocarditis, including 47 in the pandemic period and 53 in the post-pandemic period.

All hospital admissions between January 2020 and December 2025 with an ICD code for endocarditis recorded as either a primary or secondary discharge diagnosis were screened for eligibility (n = 120). Medical records were reviewed to confirm the diagnosis of infective endocarditis according to the modified Duke criteria. Twenty cases were excluded because they did not fulfil the eligibility criteria and/or had insufficient clinical information to confirm definite infective endocarditis. The final cohort comprised 100 patients with definite infective endocarditis, including 47 admitted during the pandemic period (2020–2022) and 53 during the post-pandemic period (2023–2025) (Figure 2).

Patients were stratified according to their admission period into the following groups:

Pandemic period: January 2020–December 2022.

Post-pandemic period: January 2023–December 2025.

### 4.3. Data Collection

Data were extracted from electronic medical records using a standardised form. The following variables were recorded:

- Demographic characteristics (age and sex);
- Comorbidities (including cardiovascular disease, diabetes, chronic kidney disease, and others);
- Microbiological aetiology (based on blood cultures and/or intraoperative samples);
- Valve involvement (native or prosthetic) and location;
- Echocardiographic findings, including vegetation size;
- Embolic and other complications (e.g., stroke and systemic embolism);
- Management strategy (including antimicrobial therapy and surgical intervention);
- Clinical outcomes (including in-hospital mortality and one-year survival); and
- Outcomes.

The primary outcome was in-hospital mortality. Secondary outcomes included one-year survival and the occurrence of embolic complications. Cause of death was determined from the medical records and discharge or death summaries. In-hospital deaths were classified as IE-related when death resulted directly from active infective endocarditis or from an immediate complication of IE. All deaths included in the present cohort met these criteria.

### 4.4. Statistical Analysis

Continuous variables are presented as median and interquartile range (IQR) and were compared using the Mann–Whitney U test. Categorical variables are presented as counts and percentages, and were compared using the chi-squared test or Fisher’s exact test, as appropriate.

Univariable logistic regression analysis was used to assess the association between study period and in-hospital mortality. Logistic regression was also used to assess the association between vegetation size and embolic events. Results are reported as odds ratios (ORs) with 95% confidence intervals (CIs). A two-sided *p*-value < 0.05 was considered statistically significant.

Statistical analyses were performed using SPSS Statistics version 26.0 (IBM Corp., Armonk, NY, USA). Figures were generated using Python version 3.10, utilising the Matplotlib version 3.5.2 and Seaborn version 0.11.2 for data visualisation.

### 4.5. Ethics

The study was approved by the institutional ethics committee. The requirement for informed consent was waived due to the retrospective nature of the study. All data were anonymised prior to analysis, and the study was conducted in accordance with the Declaration of Helsinki.

## 5. Conclusions

This single-centre study demonstrated that the post-pandemic period was associated with shorter diagnostic delays, lower in-hospital mortality, and improved survival compared with the pandemic period, alongside descriptive changes in the microbiological profile. These findings suggest that healthcare disruptions during COVID-19 contributed to delayed diagnosis and worse outcomes, whereas post-pandemic improvements reflect recovery and adaptation of healthcare systems. Microbiological aetiology remains a key determinant of prognosis and should guide clinical management and risk stratification.

## Figures and Tables

**Table 1:** Clinical characteristics, microbiology, complications and management of infective endocarditis during pandemic and post-pandemic periods (n = 47 and n = 53, respectively).

Variable	Pandemic (n = 47)	Post-Pandemic (n = 53)	*p*-Value
**Demographics**			
Age, years, median (IQR)	68 (61.5–72.0)	67 (58.0–74.0)	0.853
Male sex, n/N (%)	33/47 (70.2)	39/53 (73.6)	0.708
Urban residence, n/N (%)	20/38 (52.6)	26/53 (49.1)	0.737
Previous IE, n/N (%)	3/47 (6.4)	1/53 (1.9)	0.339
**Clinical presentation**			
Sepsis criteria, n/N (%)	4/46 (8.7)	6/52 (11.5)	0.746
Positive blood cultures, n/N (%)	31/43 (72.1)	33/51 (64.7)	0.444
**Type of infective endocarditis**			
Native valve IE, n/N (%)	29/47 (61.7)	33/52 (63.5)	0.857
Prosthetic valve IE, n/N (%)	16/47 (34.0)	15/52 (28.8)	0.578
Device-related IE, n/N (%)	6/47 (12.8)	6/52 (11.5)	0.852
**Type of acquisition**			
Community-acquired, n/N (%)	26/40 (65.0)	39/51 (76.5)	0.229
Healthcare-associated, n/N (%)	14/40 (35.0)	11/51 (21.6)	0.154
Mixed acquisition, n/N (%)	0/40 (0.0)	1/51 (2.0)	1.000
**Portal of entry**			
Cutaneous, n/N (%)	5/36 (13.9)	13/50 (26.0)	0.173
Digestive, n/N (%)	4/36 (11.1)	9/50 (18.0)	0.379
Oral/ENT, n/N (%)	5/36 (13.9)	11/50 (22.0)	0.340
Pulmonary, n/N (%)	1/36 (2.8)	0/50 (0.0)	0.419
Unknown portal, n/N (%)	21/36 (58.3)	19/50 (38.0)	0.062
**Aetiology**			
Staphylococci, n (%)	13 (27.7)	11 (20.8)	
Enterococci, n (%)	6 (12.8)	9 (17.0)	
Streptococci, n (%)	7 (14.9)	9 (17.0)	
Fungi (Candida), n (%)	2 (4.3)	2 (3.8)	
Other, n (%)	6 (12.8)	5 (9.4)	
Unknown (negative BC), n (%)	13 (27.7)	17 (32.1)	
**Echocardiography**			
TEE performed, n/N (%)	36/43 (83.7)	45/50 (90.0)	0.368
**Local cardiac complications**			
Any complication, n (%)	8 (17.8)	15 (30.6)	0.160
Perivalvular abscess, n/N (%)	7/47 (14.9)	12/53 (22.6)	0.324
Valve perforation, n (%)	3 (6.7)	1 (2.0)	0.346
Prosthetic dehiscence, n (%)	1 (2.2)	0 (0.0)	0.479
Prosthetic leak, n (%)	0 (0.0)	1 (2.0)	1.000
**Peripheral embolism**			
Any embolism, n/N (%)	18/46 (39.1)	33/53 (62.3)	**0.022**
Central nervous system, n/N (%)	3/47 (6.4)	16/53 (30.2)	**0.002**
Spleen, n/N (%)	6/47 (12.8)	9/53 (17.0)	0.556
Osteoarticular/discitis, n/N (%)	4/47 (8.5)	9/53 (17.0)	0.209
Kidney, n/N (%)	2/47 (4.3)	9/53 (17.0)	**0.042**
Lung, n/N (%)	2/47 (4.3)	7/53 (13.2)	0.167
**Treatment and outcomes**			
Time to diagnosis, days, median (IQR)	40 (30–54)	24 (13.8–36.3)	**<0.01**
Antibiotic duration, days, median (IQR)	42 (35–46)	42 (30–42)	0.347
Length of hospital stay, days, median (IQR)	39 (24–52)	40 (29–46)	0.932
Surgery indicated, n/N (%)	34/47 (72.3)	46/53 (86.8)	0.071
Surgery performed, n/N (%)	27/34 (79.4)	32/46 (69.6)	0.322
In-hospital mortality, n/N (%)	24/47 (51.1)	13/53 (24.5)	**0.006**
Survival, n/N (%)	23/47 (48.9)	40/53 (75.5)	**0.006**
Recurrence, n/N (%)	2/28 (7.1)	2/44 (4.5)	0.640

Data are presented as n (%) or n/N (%) for categorical variables and median (IQR) for continuous variables. Where data were incomplete, N indicates the number of patients with available observations for that variable. Continuous variables were compared using the Mann–Whitney U test and categorical variables using the chi-squared test or Fisher’s exact test, as appropriate. No *p*-value is reported for the aetiology rows. IE, infective endocarditis; TEE, transoesophageal echocardiography; IQR, interquartile range; BC, blood cultures.

## Data Availability

The data presented in this study are not publicly available due to patient privacy and ethical restrictions. De-identified data may be made available from the corresponding author upon reasonable request and with permission from the Ethics Committee, in accordance with institutional policies and applicable data protection regulations.

## Abbreviations

The following abbreviations are used in this manuscript: IE, infective endocarditis; IQR, interquartile range; TEE, transoesophageal echocardiography; CNS, central nervous system.
